# EXPERIENCES OF KOREAN GRANDFAMILIES: DIFFERENCES BETWEEN MATERNAL AND PATERNAL GRANDPARENTS

**DOI:** 10.1093/geroni/igac059.1033

**Published:** 2022-12-20

**Authors:** Youjung Lee

**Affiliations:** Binghamton University, Binghamton, New York, United States

## Abstract

Grandparent-headed families in South Korea have been growing prominent in the country’s cultural landscape. Approximately 153,000 Korean grandparent-headed households existed in 2015; this number is expected to double by 2035. This qualitative study explored Korean custodial grandparents’ experiences of raising grandchildren and the cultural significance of multigenerational caregiving in Korea. Using a phenomenological approach, semistructured interviews with 22 custodial grandparents were conducted. Significant functions of patrilineality and stigma surrounding divorce for Korean grandparent-headed families were found. Considering the complicated cultural factors, social/family service programs must pay attention to the unique needs of grandparent-headed families and consider the circumstances related to grandparents’ positions in the family (i.e., paternal vs. maternal grandparent caregivers). Korean government programs and policies could better help marginalized grandparent-headed families with an empowerment approach to help marginalized grandparent-headed families gain positive attitudes toward their caregiving situation.

